# Surface engineering of nanoporous substrate for solid oxide fuel cells with atomic layer-deposited electrolyte

**DOI:** 10.3762/bjnano.6.184

**Published:** 2015-08-27

**Authors:** Sanghoon Ji, Waqas Hassan Tanveer, Wonjong Yu, Sungmin Kang, Gu Young Cho, Sung Han Kim, Jihwan An, Suk Won Cha

**Affiliations:** 1 Graduate School of Convergence Science and Technology, Seoul National University, Iui-dong, Yeongtong-gu, Suwon 443-270, South Korea; 2 Department of Mechanical Engineering, Seoul National University, Gwanak-ro, Gwanak-gu, Seoul 151-742, South Korea; 3 Department of Mechanical Engineering, Korea Advanced Institute of Science and Technology, Daehak-ro, Yuseong- gu, Daejeon 305-701, South Korea; 4 Corporate R&D Institute, Samsung Electro Mechanics, Maeyoung-ro, Yeongtong-gu, Suwon 443-743, South Korea; 5 Manufacturing Systems and Design Engineering Programme, Seoul National University of Science and Technology, Gongneung-ro, Nowon-gu, Seoul 139-743, South Korea

**Keywords:** anodic aluminum oxide, atomic layer deposition, bottom electrode catalyst, mass transport, solid oxide fuel cell

## Abstract

Solid oxide fuel cells with atomic layer-deposited thin film electrolytes supported on anodic aluminum oxide (AAO) are electrochemically characterized with varying thickness of bottom electrode catalyst (BEC); BECs which are 0.5 and 4 times thicker than the size of AAO pores are tested. The thicker BEC ensures far more active mass transport on the BEC side and resultantly the thicker BEC cell generates ≈11 times higher peak power density than the thinner BEC cell at 500 °C.

## Introduction

Recently solid oxide fuel cells with thin film ceramic electrolytes, called thin film solid oxide fuel cells (TF-SOFCs), have drawn attention as efficient power-generation devices delivering a satisfactory power density (above 1 W/cm^2^) even below 600 °C stemming from the low ohmic resistance of thin film electrolytes [1–2]. However, their small active cell area resulted in insufficient output power, which required research on ways to enlarge the active cell area. In this regard, the use of scalable and porous substrates is one of the ways, among which anodic aluminum oxide (AAO) membranes are considered as prospective substrates due to their compatible thermal expansion properties with ceramic electrolytes, low corrosiveness, and easy manufacturing [3]. Furthermore, not containing any metallic particles, AAO substrates are advantageous on the thermo-mechanical durability compared to conventional substrate such as porous cermets [4].

Thin film electrolytes supported on porous substrates are generally vulnerable for pinhole issues causing gas permeation and electrode diffusion due to the rough surface of porous substrates [5]. This drawback necessitates conformal and dense thin film electrolytes, and can appreciably be relieved with an aid of atomic layer deposition (ALD) technique that is governed by binary reaction sequence chemistry in vacuum state [6]. Still, ALD-prepared electrolytes have rarely been utilized in porous substrate-supported SOFCs in spite of their superior characteristics; this was because the expectation that they could diminish the triple phase boundary (TPB), the meeting site between the electrolyte, the electrode, and the fuel, length and disturb the inflow of fuel at bottom electrode catalyst (BEC) side by excessive infiltration into the fuel channel [7–8]. Study on the thickness of the BEC, which could mitigate the infiltration issue, is therefore crucial in realizing the reliable TF-SOFC structure based on porous substrates such as AAO membranes.

In this study, the microstructural design of BECs in AAO supporting TF-SOFCs with ALD thin film electrolytes is discussed in terms of their impacts on the electrochemical performance of the cells. AAOs with 80 nm-sized pores are used as substrates, and the thicknesses of BECs are smaller or larger than the size of AAO pores. Although the 320 nm-thick BEC cell has slightly worse reaction kinetics, compared to the 40 nm-thick BEC cell, its peak power density is approximately 11 times higher due to far more active mass transport on the BEC side.

## Results and Discussion

### Highly dense ALD thin film electrolyte

Thin films fabricated via low-temperature vacuum deposition techniques typically have lower packing density than powder-processed thin films due to the presence of high density of grain-boundaries inside the thin films [9–10]. The density (≈5.8 g/cm^3^) of yttria-stabilized zirconia (YSZ) thin films fabricated via ALD technique is, in like manner, lower than that (≈6.1 g/cm^3^) of powder-processed YSZ [11]; nevertheless, the applied ALD process produced highly densified YSZ thin films compared to high-vacuum sputtering producing YSZ thin films with a density of ≈5.3 g/cm^3^ (i.e., sputtering has been widely used for fabricating electrolytes for TF-SOFCs).

### Cell performance and microstructural analysis

Because the AAO substrate used as a supporter for membrane electrode assembly is non-conductive and catalytically inactive, BEC needs to be coated on the AAO substrate prior to the electrolyte deposition for the anode side current collection and catalytic reaction. We considered the reaction kinetics at the BEC–electrolyte interface and fuel transport through AAO pores as the main design parameters in BEC coating.

**Figure 1:**
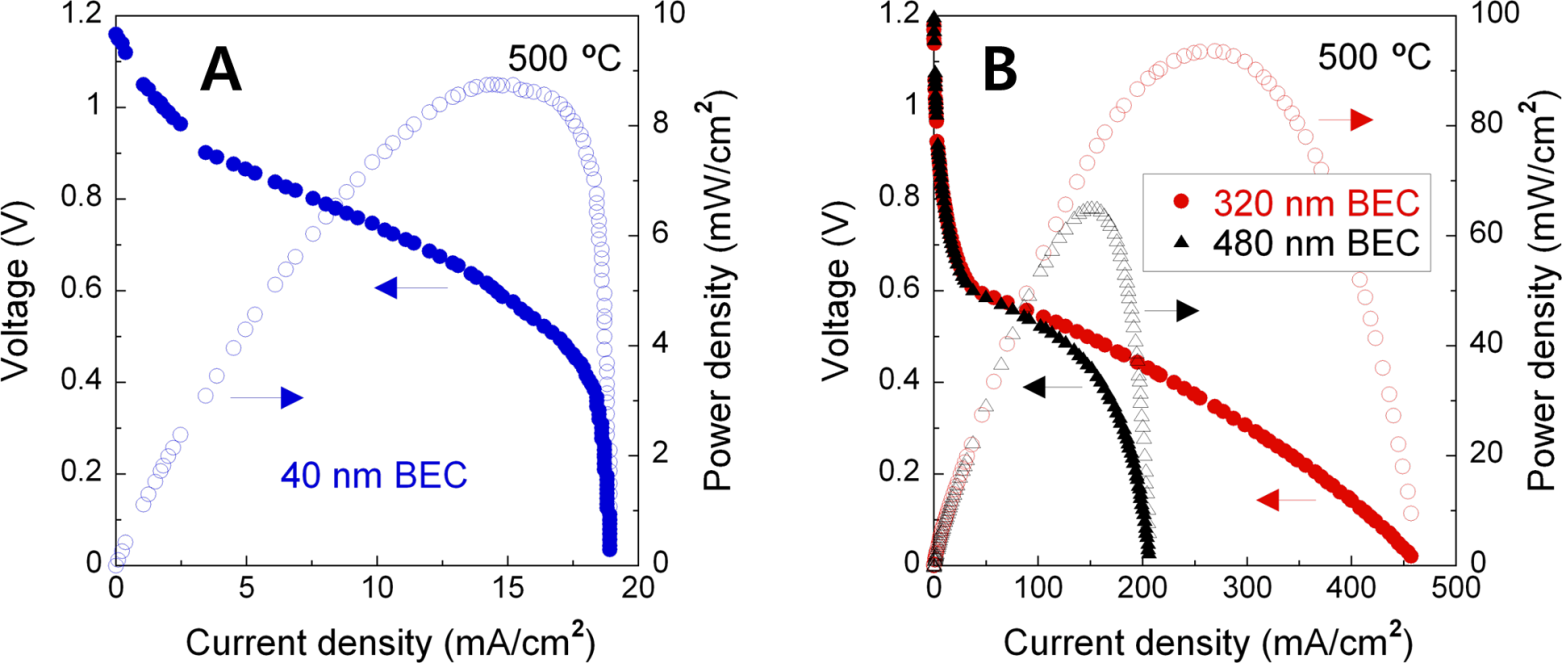
*I*–*V* and power density curves, measured at 500 °C, for 80 nm pore AAO supporting (A) 40, (B) 320 and 480 nm-thick bottom electrode catalyst (BEC, sputtered Pt anode deposited under high-vacuum) solid oxide fuel cells, referred to the Cell-A, Cell-B and Cell-C, having 210 nm-thick atomic layer-deposited (ALD) yttria-stabilized zirconia (YSZ) electrolyte and 60 nm-thick top electrode catalyst (sputtered porous Pt cathode).

To investigate the effects of BEC thickness on the electrochemical performance, polarization curves were plotted for 40, 320, and 480 nm-thick BEC cells having 210 nm-thick ALD YSZ electrolyte and 60 nm-thick top electrode catalyst (used as cathode), referred to the Cell-A, Cell-B, and Cell-C (Figure 1). Three kinds of cells generates high open circuit voltages (OCVs) of ≈1.17 V implying the high integrity of conformal and dense YSZ electrolytes, which is quite contiguous to the theoretical OCV value of 1.18 V under the operating conditions [12]. However, the overall voltage drop of the Cell-A with increasing current density is much bigger than that of the Cell-B, which results in a ≈11 times lower peak power density (8.8 mW/cm^2^) compared to that of Cell-B (93.1 mW/cm^2^). In particular, as shown in Figure 1A, the voltage drop of the Cell-A at high current densities (near ≈19 mA/cm^2^) is considerably sharp, which means that the Cell-A suffers from serious concentration loss compared to the Cell-B [13]. Owing to the prominent voltage drop at current densities above 100 mA/cm^2^, the peak power density (65.3 mW/cm^2^) of the Cell-C was somewhat smaller than that of the Cell-B (Figure 1B). This performance reduction may be due to the impoverished mass transport and shortened TPB length caused by excessively thick BEC, which is parallel to previous research discussing the effects of the thickness and microstructure of BECs [14].

**Figure 2:**
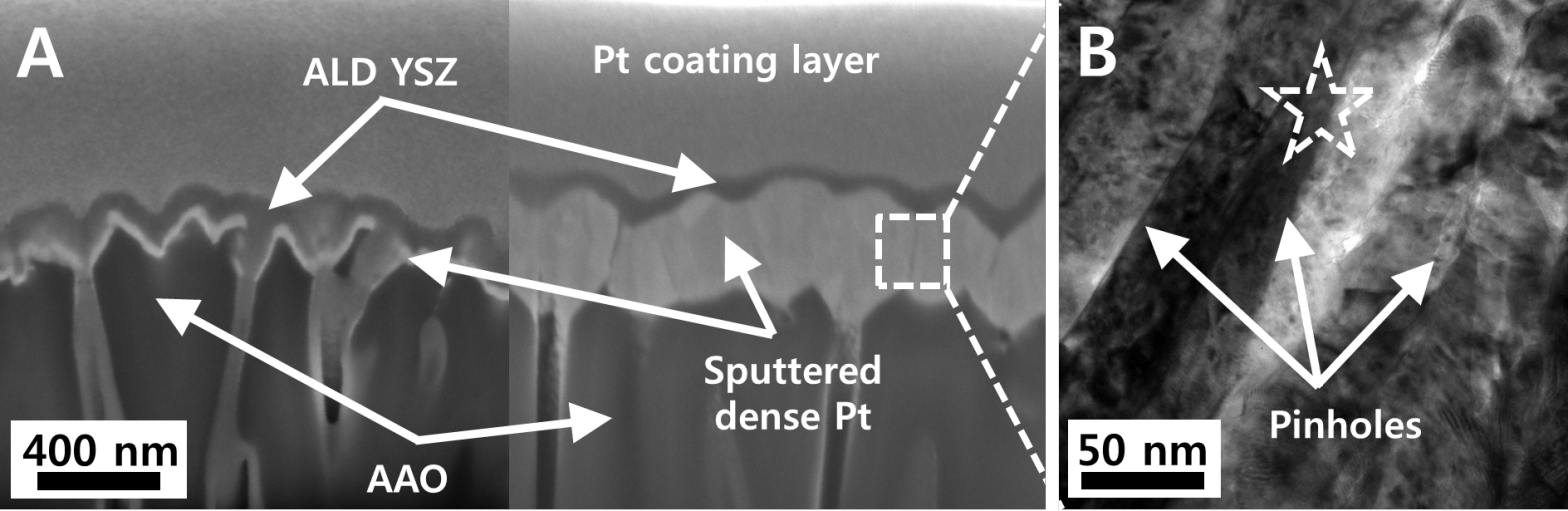
(A) Focused ion beam-prepared field emission scanning electron microscopy (FE-SEM) cross-sectional images for 50 nm-thick ALD YSZ films deposited on 80 nm pore AAO supported 40 (left side) and 320 (right side) nm-thick BECs; (B) transmission electron microscopic image for 80 nm pore AAO supported 320 nm-thick BEC.

To examine the diffusion characteristics of ALD YSZ on the BEC side, 50 nm-thick ALD YSZ was deposited on BECs with different thicknesses, whose cross-sectional microstructure was investigated by focused ion beam and field emission scanning electron microscopy (FIB/FE-SEM) imaging: the BECs were 40 nm and 320 nm in thickness. In case of the thinner BEC, a significant amount of ALD YSZ certainly infiltrates into the interior of the BEC as well as into AAO pores (the left image of Figure 2A), which may have negative impacts on fuel supply through AAO pores. In case of the thicker BEC, on the other hand, most of the conformal YSZ is deposited on the top surface of the BEC, as shown in the right image of Figure 2A. The thicker BEC could remarkably alleviate the infiltration of ALD YSZ into the interior of AAO pores. This pronounced difference in infiltration aspect of ALD YSZ should be closely linked to growth characteristics of sputtered films [12]. The thickness increase of physical vapor-deposited (PVD) films deposited on AAO pores expands their column-width and reduces the size of pinholes (or voids) existing in the sputtered films. We thus think that the merging of columnar grains of BEC according to the thickness increase lowers the infiltration degree of ALD YSZ into the BEC and AAO pores. This consideration is parallel to the interpretation from the *I*–*V* analysis result of Figure 1 discussed in the previous section. Meanwhile, the existence of a few nanometer-sized pinholes formed throughout the thicker BEC, which could provide the physical space to diffuse H_2_ gas supplied to the anode side, implies the possibility of TPB formation on the BEC side (Figure 2B). The transmission electron microscopy and energy-dispersive X-ray (TEM-EDX) quantitative analysis result in the middle of the thicker BEC (at dotted asterisk) verified the constituent elements of Pt (78.9%), Zr (6.9%), Y (0.5%), and O (13.7%), meaning that such pinholes were filled by the ALD YSZ.

**Figure 3:**
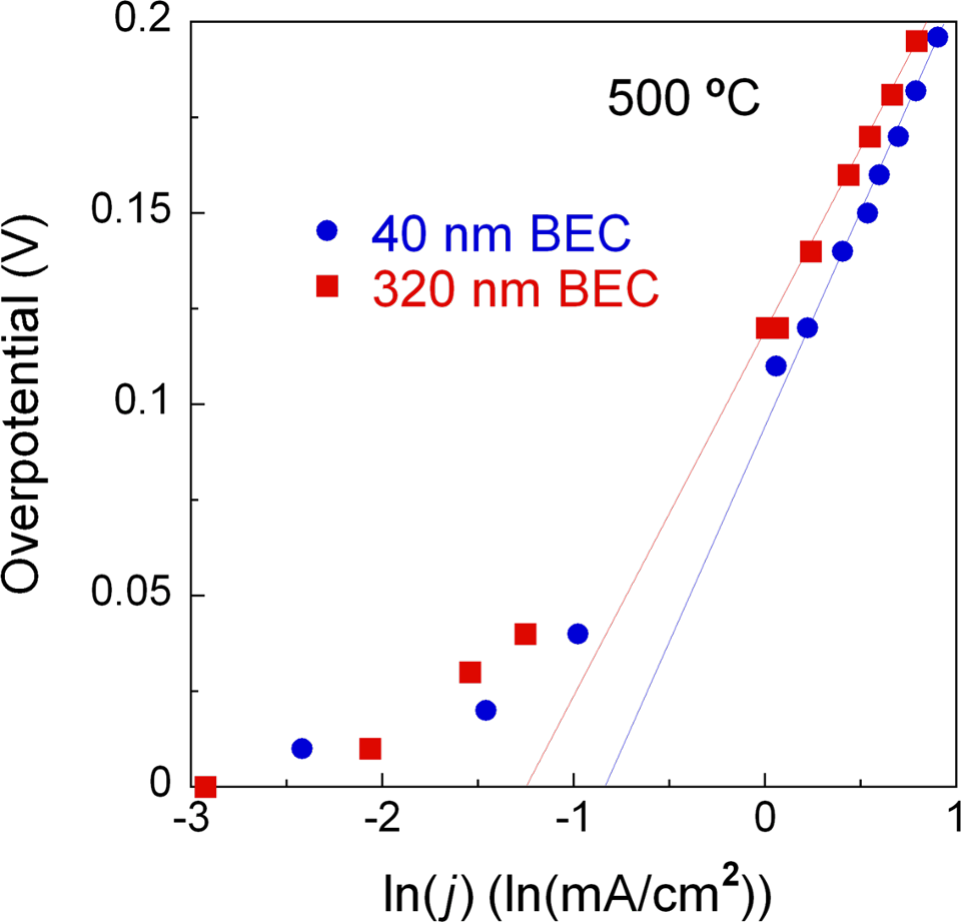
Tafel plots, measured at 500 °C, for the Cell-A and Cell-B.

Interestingly, the onset point of a voltage plateau for the Cell-B was as low as 0.6 V contrary to that of conventional SOFCs. This phenomenon is likely due to the remarkably large activation loss compared to other kinds of losses; the possible reasons for this deactivation are the insufficient electrocatalytic activity of the Pt BEC and the lack of TPB at the electrode–electrolyte interface [15–16]. The exchange current densities obtained by Tafel fitting were 0.43 mA/cm^2^ and 0.29 mA/cm^2^ for the Cell-A and Cell-B, respectively, as shown in Figure 3 [17]. Although the values were not significantly different each other, this fitting result indicates that the Cell-A may have somewhat longer TPB length at the BEC side and therefore faster reaction kinetics than the Cell-B, based on the interpretation described in related research [18–19]. One speculated reason of the longer TPB length for the Cell-A is that more infiltrated ALD YSZ electrolyte into the thinner BEC could have larger BEC–electrolyte contact area, referring to the cross-sectional FE-SEM imaging result of Figure 2A, than the counterpart.

Consequently, the performance comparison and microstructural analysis imply that the thicker BEC elicits higher peak power density due to the superior mass transport through the pores of the AAO substrate in spite of the slightly slower reaction kinetics at the BEC–electrolyte interface.

### Measurements of individual resistances via impedance spectroscopy

**Figure 4:**
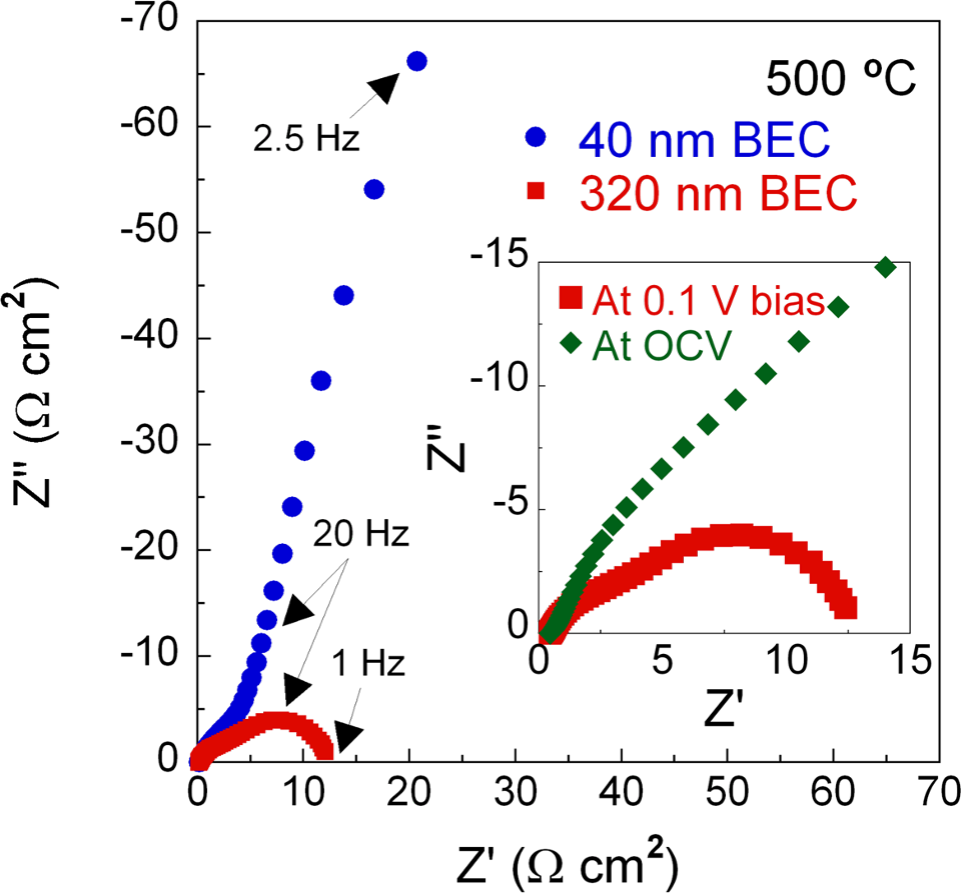
Electrochemical impedance spectroscopy analysis results, measured at 500 °C, at bias voltage of 0.1 V for the Cell-A and Cell-B, and (inset) at open circuit voltage and bias voltage of 0.1 V for the Cell-B.

To investigate the effects of BEC thickness on the individual resistances, electrochemical impedance spectroscopy (EIS) data were obtained for the Cell-A and Cell-B. Before comparing the EIS data for two kinds of cells, the EIS curves obtained under different direct current (DC) bias voltages (OCV and 0.1 V with respect to the cathode) for the Cell-B were overlapped to differentiate the ohmic resistance (resulting from charge transport inside electrolyte) from the activation resistance (resulting from reaction kinetics at electrode–electrolyte interface), as shown in the inset of Figure 4 [20]. The comparison result indicates that all of the semicircles are relevant to the activation process, i.e., electrode–electrolyte interfacial resistance, not to the ohmic process, i.e., electrolytic resistance, because there are no overlapping semicircles. Figure 4 shows EIS curves obtained under a DC bias voltage of 0.1 V for the Cell-A and Cell-B. The EIS curve for the Cell-B contains two predominant semicircles with peak imaginary values at 1 kHz and at 20 Hz by a non-linear least square fitting to the equivalent circuit consisting of one resistance (related to ohmic resistance) and two pairs of constant phase element and resistance (related to electrode–electrolyte interfacial resistance) [6]. Referring to the previous literatures [6,20–22], it is considered that semicircles at higher and lower frequencies correspond to the anode and cathode interfacial resistances, respectively. The Cell-A, on the other hand, shows the EIS behavior with a diagonal form at a lower frequency region below 20 Hz, which is not observed in the impedance spectra of Cell-B. This diagonal component is considered to the effect of Waburg element signifying a lack of active fuel supply [13]. This interpretation corresponds well to the above-mentioned polarization analysis, where we observed a sharp drop in the cell voltage of Cell-A at *j* > ≈19 mA/cm^2^. The different shape of the semicircle around 20 Hz – which is regarded as the frequency time constant of cathode interfacial resistance [1] – of Cell-A compared with that of Cell-B seems to be due to the overlap of the cathode loop and the Warburg element. The high frequency intercept that corresponds to ohmic resistance is 0.5 and 0.45 Ω·cm^2^ for the Cell-A and Cell-B, respectively. Considering both cells have similar cathode sheet resistance of 540–560 Ω·cm and the electrolyte thickness is about the same, such a difference in ohmic resistance may be attributed to a difference in anode sheet resistance stemming from the different thicknesses. Nevertheless, this slight difference in ohmic resistance is immaterial to the peak power density of two cells (e.g., a voltage difference between the Cell-A and Cell-B is only 0.5 mV at 10 mA/cm^2^ that seems to be the range where the ohmic loss becomes dominant). Consequently, we think that the mass transport at the anode side needs to be considered as a dominant factor to determine the performance of AAO-supported TF-SOFCs with ALD thin film electrolyte as well as reaction kinetics and ohmic performance.

## Experimental

### Thin film fabrication

ALD YSZ film was deposited with a showerhead-type plasma-enhanced ALD machine (Atomic Premium, CN1, South Korea) capable of accommodating one six-inch wafer with a radio frequency plasma generator. The processing chamber with a load-lock wafer handler was vacuumized using a dry pump to a base pressure of 2.7 Pa. The temperature of the sample stage was set to 250 °C. The detailed fabrication process of the YSZ film is presented in our previous work [23]. PVD YSZ and Pt films were deposited with a sputtering machine (A-Tech System, South Korea) equipped with a custom-designed rotating unit ensuring the high thickness uniformity; the rotating unit was revolved at 4 rpm. The target-to-substrate distance was 75 mm, and the substrate was not heated. For deposition of the YSZ film, a gas mixture of Ar and O_2_ in the volumetric ratio of 80:20 was used. Background pressure was kept at 1.3 Pa during deposition. Radio frequency magnetron power of a sputtering gun was set to 50 W. A two inch-sized YSZ disk pellet with an 8 mol % Y_2_O_3_ was used as the target. For deposition of the Pt film, 99.99% purity Pt disk was used as the target. Porous Pt film (for cathode) and much denser Pt film (for anode, BEC) were deposited at 12 Pa and 0.7 Pa, in an Ar gas atmosphere, respectively. The DC power of a sputtering gun was set to 200 W, and the purity of Ar gas was 99.99%. The fabrication processes of the Pt films are close to the ways described in our preview work [24].

### Thin film characterization

The film density was determined by X-ray reflectometry analysis using the X’Pert Pro (PANalytical, Netherlands) instrument. The surface microstructure was investigated by FIB/FE-SEM analysis using the quanta 3D FEG (FEI Company, Netherlands) instrument. Local surface composition was measured by TEM-EDX analysis using the JEOL-2100F (JEOL, Japan) instrument. The characterization techniques utilized in this study are close to the measures described in our preview work [23].

### Electrochemical evaluation

**Figure 5:**
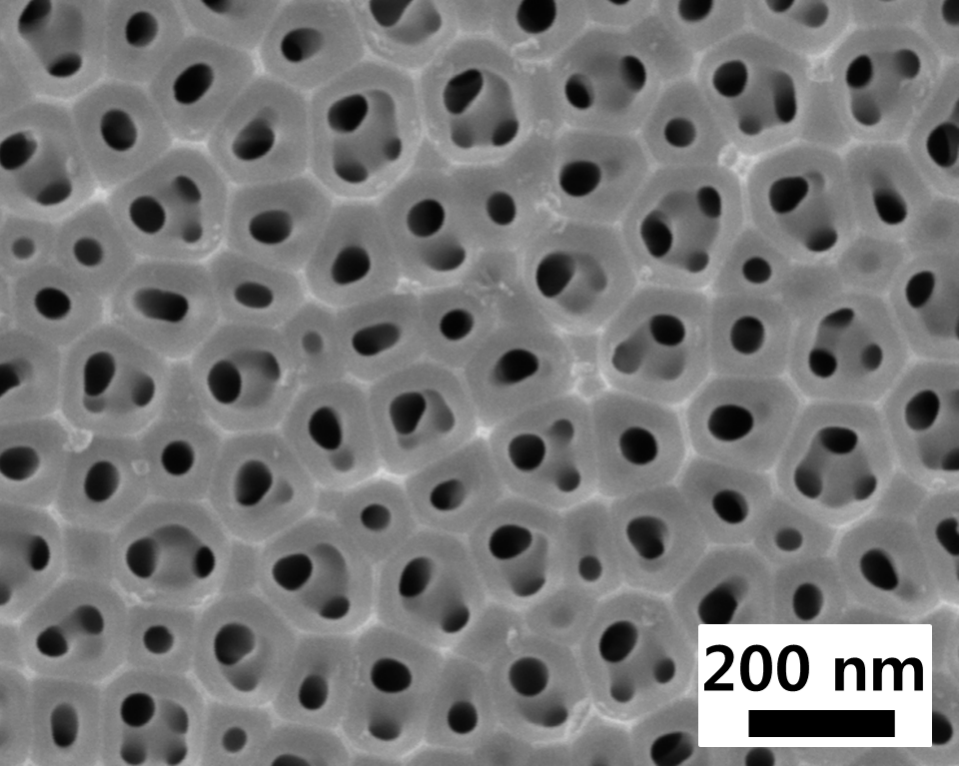
FE-SEM top-view image of AAO membrane with well-arrayed 80 nm-sized pores, cleaned by sonication in ethanol.

Commercial AAO (Synkera, USA) membrane with the thickness of 100 μm and the pore size of 80 nm, as shown in Figure 5, was used as the porous substrate to support TF-SOFCs. Test cells with an active electrode area of 1 mm^2^ were attached to the custom-designed gas feeding chamber using a ceramic adhesive (CP4010, Aremco Products, USA), which were heated to 500 °C with a ramping rate of 10 °C/min using halogen heaters. 50 sccm dry H_2_ gas was supplied to the anode side and the cathode was exposed to the atmospheric environment. The anode was connected with a combination of silver paste (597A, Aremco Products, Inc., USA) and a 0.5 mm-thick silver wire, while the cathode was contacted using a hardened-steel tip with a radius of 0.19 mm probe moved by a XYZ stage. Electrochemical characterization was performed in the two-electrode configuration without a reference electrode. EIS analysis was carried out using an electrochemical testing system (1287/1260, Solatron Analytical, UK), in which the alternating current amplitude was set to 50 mV. The testing method is close to the way presented in our preview work [6].

## Conclusion

We discussed about the preliminary design of BEC for porous AAO supporting TF-SOFCs with conformal and dense thin film electrolyte prepared by ALD technique. The thickness of BEC had a significant impact on the infiltration degree of ALD electrolyte into the BEC and the AAO substrate. The infiltration degree of ALD electrolyte moderated when the thicker BEC was employed, which led to the generation of appreciably higher peak power density caused by more active mass transport on the BEC side. Such thicker BEC improved current collecting performance to some degree; however, resulted in slightly slower reaction kinetics. Further optimization of BEC thickness may enhance the cell performance, which could lead to wider potential applications of AAO supporting TF-SOFCs as high-efficiency power sources. In addition, the discussion presented in this paper may help to design high-performance porous substrate-supported TF-SOFCs with few hundred nanometer-thick BECs.
